# Nitrogen Type and Availability Drive Mycorrhizal Effects on Wheat Performance, Nitrogen Uptake and Recovery, and Production Sustainability

**DOI:** 10.3389/fpls.2020.00760

**Published:** 2020-06-19

**Authors:** Rosolino Ingraffia, Gaetano Amato, Moisés A. Sosa-Hernández, Alfonso S. Frenda, Matthias C. Rillig, Dario Giambalvo

**Affiliations:** ^1^Department of Agricultural, Food and Forest Sciences, Università degli Studi di Palermo, Palermo, Italy; ^2^Plant Ecology, Institute of Biology, Freie Universität Berlin, Berlin, Germany; ^3^Berlin-Brandenburg Institute of Advanced Biodiversity Research, Berlin, Germany

**Keywords:** arbuscular mycorrhizal fungi, arbuscular mycorrhizal (AM) symbiosis, soil nitrogen (N) source, soil nitrogen (N) availability, organic nitrogen, mineral nitrogen, nitrogen uptake, ^15^N fertilizer recovery

## Abstract

Plant performance is strongly dependent on nitrogen (N), and thus increasing N nutrition is of great relevance for the productivity of agroecosystems. The effects of arbuscular mycorrhizal (AM) fungi on plant N acquisition are debated because contradictory results have been reported. Using ^15^N-labeled fertilizers as a tracer, we evaluated the effects of AM fungi on N uptake and recovery from mineral or organic sources in durum wheat. Under sufficient N availability, AM fungi had no effects on plant biomass but increased N concentrations in plant tissue, plant N uptake, and total N recovered from the fertilizer. In N-deficient soil, AM fungi led to decreased aboveground biomass, which suggests that plants and AM fungi may have competed for N. When the organic source had a low C:N ratio, AM fungi favored both plant N uptake and N recovery. In contrast, when the organic source had a high C:N ratio, a clear reduction in N recovery from the fertilizer was observed. Overall, the results indicate an active role of arbuscular mycorrhizae in favoring plant N-related traits when N is not a limiting factor and show that these fungi help in N recovery from the fertilizer. These results hold great potential for increasing the sustainability of durum wheat production.

## Introduction

Durum wheat is a keystone crop in Mediterranean agroecosystems. Its performance is strongly dependent on the availability of soil nitrogen (N) throughout its development. Evidence from field experiments points to a drastic decrease in crop yield with a decrease in the amount of N fertilizer (Gao et al., 2009; Giambalvo et al., 2018). However, often only 50% or less of the N fertilizer applied to soil is recovered by cereals, and this percentage decreases as the rate of N fertilization increases (Foulkes et al., 1998; Raun and Johnson, 1999; Blankenau et al., 2002; Ruisi et al., 2015, 2016). This has important agro-environmental implications since, due to the high N mobility in the soil–plant–atmosphere system, N not used by plants contributes greatly to agriculture-related pollution through leaching, volatilization, and denitrification (Drinkwater et al., 1998; Limaux et al., 1999).

Arbuscular mycorrhizal (AM) fungi can play an essential role in the plant’s acquisition of N from both organic and inorganic sources (Johansen et al., 1994; Hawkins et al., 2000; Saia et al., 2014; Thirkell et al., 2016). Various molecular studies have further corroborated that expression of plant N transporters can be influenced by AM fungi (Guether et al., 2009a, b; Saia et al., 2015), suggesting that mycorrhization might have an active influence in this process. The increase in plant N uptake due to AM symbiosis and the amount of N immobilized in fungal biomass can drastically reduce the amount of nutrients lost through different pathways in the agroecosystem (Asghari and Cavagnaro, 2012; Cavagnaro et al., 2015; Köhl and van der Heijden, 2016). The preferential uptake and immobilization of ammonium into fungal biomass reduces the amount of N that can be nitrified and lost *via* leaching, as nitrate is highly mobile in soil and easily leached. This reduction in nitrification in turn reduces the production of nitrous oxide (Bender et al., 2014; Zhang et al., 2015; Storer et al., 2018), a potent greenhouse gas with a warming potential approximately 300 times higher than that of carbon dioxide (IPCC, 2001). In addition, AM fungi can influence the soil microbial community and soil aggregation and therefore processes such as mineralization, nitrification, and denitrification (Veresoglou et al., 2012; Leifheit et al., 2014; Cavagnaro et al., 2015). Moreover, because a significant amount of the total AM fungal biomass can be located in deeper layers of the soil (Higo et al., 2013), as highlighted by Sosa-Hernández et al. (2019), a portion of N that migrates down the profile can be immobilized in the fungal biomass or delivered to the plant, thus further avoiding N loss. This notwithstanding, the role of AM fungi in N plant nutrition is controversial (Smith and Smith, 2011; Corrêa et al., 2015; Wang et al., 2018). AM fungi have a notable N demand for their metabolism (Hodge and Fitter, 2010) and can even compete with the host plant for soil N when the soil is deficient in N (Treseder and Allen, 2002; Püschel et al., 2016). Indeed, certain studies show that soil N deficiency induces the expression of N-retaining ammonium transporters in AM fungi, suggesting the existence of a competition between plant and fungus for the interface apoplast N (Guether et al., 2009b; Pérez-Tienda et al., 2011). Nonetheless, various AM fungal taxa have different functional responses to soil N availability and thus differentially affect plant and agroecosystem productivity (Treseder et al., 2018). Also, the small diameter of the AM fungal extra-radical mycelium facilitates the exploration of soil not accessible to plant root hairs, and hyphae can take up a portion of N retained by the exchange complex in the form of ammonium. This allows the two symbionts to acquire N from different portions of the soil, which can reduce competition between the plant and fungi. Moreover, although AM fungi can enhance mineralization by adding carbon (C) to sites where mineralization takes place (Hodge and Storer, 2015; Bunn et al., 2019) and/or by influencing the soil microbial community (Veresoglou et al., 2012), it is not known whether this ability changes with the composition of organic matter (e.g., different C:N ratios). In fact, during the mineralization of organic matter with a high C:N ratio, there can be a temporary immobilization of N (Tian et al., 1992) and thus a reduction in soil N availability with possible consequences for AM symbiotic functioning.

The present experiment tested whether AM fungi enhance durum wheat N uptake and N recovery from added mineral fertilizer and organic matter and whether these effects differ with varying N availability and properties of the added organic matter.

## Materials and Methods

### Pot and Plant Management

Durum wheat (*Triticum durum* Desf. cv. Anco Marzio) was grown in 6-L sterilized pots (diameter = 16 cm, height = 30 cm) filled with sterilized artificial substrate. The growth substrate was composed of a 70% silica sand (Gras Calce srl, Trezzo sull’Adda, Italy) and 30% agricultural soil w/w mixture; we used such high percentage of silica sand both to have a substrate poor in N and to easily extract all the roots. Both substrate portions were sieved through a 2-mm mesh and sterilized as follows: humidification, 24 h at room temperature, and 24 h at 130°C, for a total of three cycles. After the sterilization, the two soil fractions were separately characterized. Sand total N (Kjeldahl) and available phosphorous (P; Olsen P) were 0.11 g kg^–1^ and 7.44 mg kg^–1^, respectively. Agricultural soil was collected from the first 30 cm of a well-structured clay soil classified as a Vertic Haploxerept at Pietranera farm (Sicily, Italy; 37°53′ N, 13°51′ E; 162 m a.s.l.) having the following properties: 267 g kg^–1^ clay, 247 g kg^–1^ silt, 486 g kg^–1^ sand, pH 8.0, 10.8 g kg^–1^ total C (Walkley–Black), 0.86 g kg^–1^ total N (Kjeldahl), 40.1 mg kg^–1^ available P (Olsen P), 598 mg kg^–1^ total P, 26 cmol kg^–1^ cation exchange capacity, 1.70 dS m^–1^ saturated electrical conductivity (25°C), 27.9% water content at field capacity, and 18.9% water content at the permanent wilting point. Therefore, the resulting mixture was poor in N and sufficiently supplied with phosphorus.

Each pot was filled with 7.5 kg substrate. The crop was sown on February 3, 2016, with 15 surface-sterilized seeds per pot. Ten days after emergence, plants were thinned to seven plants per pot. All pots and seeds were sterilized with sodium hypochlorite 3% for 3 to 5 min. After sowing, all pots were irrigated to water holding capacity. Afterward, soil moisture was monitored twice a week, and additional water was added when the soil moisture reached 70% of water holding capacity. The soil water holding capacity of the substrate was determined with the gravimetric method (Dobriyal et al., 2012). Briefly, 10 perforated crucibles were filled with 100 g soil and placed in a basin with water up to half of the crucibles’ height. The crucibles were permitted to absorb water by capillarity until each pot was saturated. Excess water was allowed to drain, and the crucibles were weighed and oven-dried at 105°C to a constant weight. The weight difference between the crucibles before and after the drying process represented the soil water content at field capacity.

The factors studied were fertilization (five levels: non-fertilized control, two levels of mineral N supply, two organic matter amendments) and inoculation (two levels: non-inoculated control, AM fungal inoculation). A total of 50 pots were set up [2 (with or without AM fungal inoculum) × 5 soil N levels × 5 replicas] in a completely randomized design. The plants were grown for 85 days after sowing (DAS), from February 3 to April 28, 2016. The experiment was performed outdoors at Pietranera farm, which is located about 30 km north of Agrigento, Sicily, Italy (37°54′ N, 13°51′ E; 160 m a.s.l.). Weather data collected from a weather station within 200 m of the experimental location are reported in Supplementary Figures S1, S2.

### Fertilization Treatments

Durum wheat in the presence (+myc) or absence (–myc) of AM fungal inoculum was grown under four N fertilizer treatments (N-org1, N-org2, N-min1, N-min2) and in a non-fertilized treatment (N0). Briefly, 10% ^15^N-enriched ammonium sulfate was applied in N-min1 and N-min2. In N-min1, 0.75 g fertilizer was applied per pot. In N-min2, the amount of N was doubled; hence, 1.5 g fertilizer per pot was added. In the two mineral treatments, there were two fertilization events: two thirds of the total fertilizer (0.5 and 1 g per pot in N-min1 and N-min2, respectively) was applied 11 days after emergence, and the remaining one third (0.25 and 0.5 g per pot, respectively) was applied 38 DAS (concomitant with the beginning of the durum wheat elongation phase).

All organic N treatments consisted of the application of 13 g organic matter per pot (equivalent amount of 6.5 Mg ha^–1^). This amount is approximately equivalent to the typical amount of biomass left by the previous crop in the field in this semiarid agroecosystem (Giambalvo et al., 2012).

The organic matter was chopped (approximately 2 mm) and homogeneously distributed at a depth of 5 to 10 cm 1 day before sowing. Residues of two crops with different C:N ratios, *Lolium multiflorum* (ryegrass; N-org1) and *Vicia faba* (faba bean; N-org2), were used as organic N sources. The organic matter for both treatments was obtained from a pilot experiment in which the two species were grown in ^15^N-enriched soil. The pilot experiment ended when both species reached maturation. Later, the biomass was harvested, dried, and characterized for total C, N, and ^15^N concentrations (Table 1). Characteristics of the organic matter are reported in Table 1. In short, 0.118 and 0.363 g organic N per pot were applied in N-org1 and N-org2, respectively.

**TABLE 1:** Properties of the organic nitrogen (N) sources used in the experiment.

**Crop**	**Total C**	**Total N**	**C to N**	**^15^N**
**residual**	**(g kg** ^–^**^1^ dry**	**(g kg** ^–^**^1^ dry**	**ratio**	**(g kg** ^–^**^1^ total N)**
	**weight)**	**weight)**		
Ryegrass	453	9.1	49.7	8.9
Faba bean	447	27.9	16.2	4.3

*Ryegrass and faba bean were used in N-org1 and N-org2, respectively.*

### Inoculum

At sowing time, the natural soil microbial community, excluding AM fungi, was reintroduced to each pot. To this end, a soil filtrate was obtained through filtration of a soil suspension. Briefly, soil was suspended in distilled water at a ratio of 1:3 w/v and shaken for 20 min at 140 rpm. Later, after decantation, the suspension was filtered through an 11 μm mesh to remove the natural AM fungal community. A total of 200 ml soil filtrate solution was added per pot. Pots in the +myc treatments were also inoculated with 1 g per pot of a commercial inoculum (AEGIS IRRIGA, Italpollina SpA, Rivoli Veronese, Italy) consisting of a mix of the two AM fungi species Rhizophagus irregularis and Funneliformis mosseae equally present at a density of 700 spores g^–1^. This commercial inoculum also had 1 × 10^7^ rhizosphere bacteria. To isolate the effects of the AM fungi, we extracted the bacterial community of the inoculum using the same protocol used for the natural soil microbial community reported above and introduced to the –myc treatments.

Arbuscular mycorrhizal fungal inoculation was done at the time of sowing, and the inoculum was distributed just below the sowing bed.

### Biomass Harvesting and Analyses

At the end of the experiment (84 DAS), aboveground biomass (shoots) was harvested and fresh and dry biomass recorded. Later, aboveground biomass was ground to a fine powder, and total N and ^15^N concentrations were determined. The total N concentration was determined with the Dumas method (flash combustion with an automatic N analyzer; DuMaster D-480, Büchi Labortechnik AG, Flawil, Switzerland), whereas the ^15^N concentration was determined using an elemental analyzer (Model NA 1500, Carlo Erba, Milan, Italy) equipped with an isotope ratio mass spectrometer (Isoprime Ltd., Cheadle, United Kingdom).

Belowground biomass (roots) was carefully extracted through sieving and consecutive washing and then oven-dried at 40°C until a constant weight.

Two root biomass subsamples were extracted. One was used to quantify the root length using the modified Newman formula (Tennant, 1975):

Root⁢length=(11/14)×N×G,

where N is the total number of intercepts of the root with vertical and horizontal grid lines, and G is the grid square dimensions (cm).

The other root subsample was cleared with potassium hydroxide 10%, stained with trypan blue 0.05% (Phillips and Hayman, 1970), and used to quantify the percentage of AM fungal infection using the method proposed by McGonigle et al. (1990). We assayed the AM fungal infection at 400× magnification by scoring a minimum of 150 intersects for the presence of intra-radical AM fungal structures.

### Soil Sampling and Analyses

During the root extraction, a representative soil sample was collected. The soil sample was sieved at 2 mm and immediately stored at −20°C to minimize changes in nutrients. Later, we assayed soil mineral N (nitrate, nitrite, and ammonium) content at the sampling stage through a colorimetric method using the Bran+Luebbe GmbH AutoAnalyzer 3 (Norderstedt, Germany). Briefly, 10 g soil was extracted in 100 ml of a 2 M potassium chloride-extractable solution and shaken for 1 h at 140 rpm. The solution was later filtered through filter paper (Whatman 42) and used to assay N-NH_4_^+^, N-NO_3_^–^, and N-NO_2_^–^ concentrations.

### Calculations and Statistical Analyses

The total root length was calculated based on the known weight of both the subsample and the total root biomass. We calculated the specific root length by dividing the total root length by the total root weight, and we obtained the soil root density by dividing the total root length by the amount of soil in the pot (grams of soil per pot).

We obtained the N uptake by multiplying the N concentration of the aboveground biomass with the amount of aboveground biomass harvested in each pot. The ^15^N concentration was used to determine the amount (^15^N_*rec*_) and percentage (%N_*rec*_) of N recovered from the fertilizer, respectively, with Eqs 1 and 2 according to Hauck and Bremner (1976):

(1) Nr⁢e⁢c15=Nu⁢p⁢t×Nf⁢p15-15Nn⁢f⁢pNf⁢e⁢r⁢t15-15Nn⁢f⁢p

(2) %⁢Nr⁢e⁢c=Nr⁢e⁢c15f×100

where *^15^N_*fp*_* is the ^15^N atom% in the fertilized plant, ^1^*^5^N_*nfp*_* is the ^15^N atom% in the non-fertilized plant (N0) from the same inoculation treatment, *^15^N_*fert*_* is the ^15^N atom% in the fertilizer, and *f* is the fertilizer rate (g pot^–1^).

A two-way factorial analysis of variance (ANOVA) was used to determine the effects of N treatment, AM fungal inoculation, and their interaction. The analyses were performed with R version 3.6.0 (R Core Team, 2019). Shapiro and Bartlett tests were used to assess the normality and homoscedasticity, respectively, of the model residuals. When response variables did not fulfill the ANOVA assumptions, the data were transformed accordingly. Following the ANOVA, pairwise comparisons using the “emmeans” package (Lenth, 2019) and confidence intervals using the “dabestr” package (Ho et al., 2019) were used to investigate the effects of mycorrhization within each fertilization treatment. All *p*-values derived from pairwise comparisons and confidence intervals are reported in tables and figures, as recommended by Gardner and Altman (1986). This method was used to avoid the problem of *p-values* dichotomous cutoffs (Wasserstein and Lazar, 2016; Betensky, 2019; Wasserstein et al., 2019).

Correlations between the percentage of root colonization by AM fungi and N applied (fertilizer N concentration × amount of fertilizer per pot) were calculated using the inoculated treatments.

Non-transformed data are reported in tables and figures. The “tidyverse” package (Wickham, 2017) was used to represent the data graphically.

## Results

### Mycorrhizal Colonization

**FIGURE 1:**
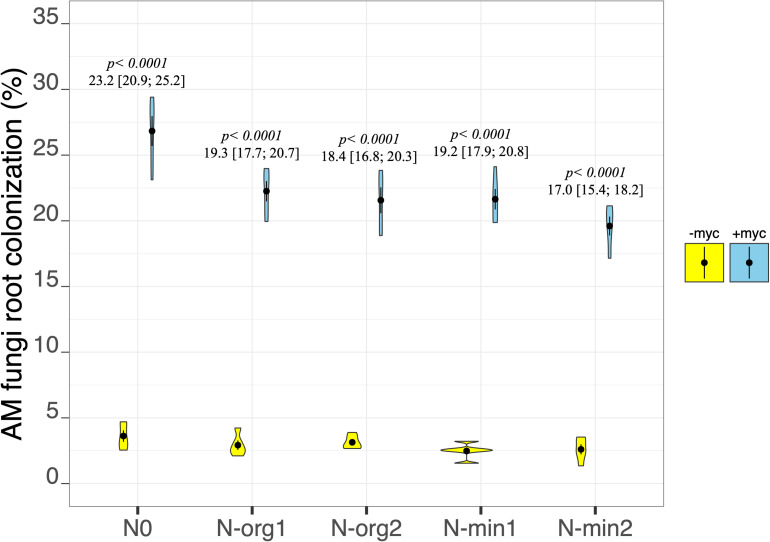
Arbuscular mycorrhizal (AM) fungi root colonization of durum wheat in the different fertilization treatments in the absence (–myc) or presence (+myc) of AM fungal inoculum. Circles inside plots represent means, with whiskers representing ± SE (*n* = 5). The width of the plot shows the density distribution of values. *p*-values for pairwise comparisons, Δ means, and estimated 95% confidence intervals (CIs) in brackets between +myc and –myc within the same fertilization treatment are reported above the plots.

Although AM fungal colonization was observed in the non-inoculated treatments (–myc), the extent of the colonization was always less than 4%, very different from the values observed in the inoculated treatments (+myc; AM fungal inoculation treatment *p* < 2e^–16^; Figure 1 and Table 2). The percentage of colonization in the inoculated treatments ranged from an average of 19.60% ± 0.72% in N-min2 to an average of 26.83% ± 1.13% in N0 (Figure 1). The percentage of AM fungal colonization was higher in N0 than all other treatments, although moderate differences were observed among all other treatments. Overall AM fungal colonization was negatively correlated with the amount of N applied (*r* = –0.66, *p* = 0.0004).

**TABLE 2:** ANOVA results for arbuscular mycorrhizal (AM) fungi root colonization, aboveground biomass dry weight, belowground biomass dry weight, nitrogen (N) uptake, and N recovery.

**Source**	***df***	**AM fungi colonization (%)**	**Aboveground biomass**	**Belowground biomass**	**Nitrogen uptake**	**N recovery**
**Fert**	**4**	*p* = 0.0001	*p* < 2e^–16^	*p* = 9.85e^–15^	*p* < 2e^–16^	*p* < 2e^–16^
**Myc**	**1**	*p* < 2e^–16^	*p* = 0.1586	*p* = 0.4090	*p* = 0.1090	*p* = 5.96e^–11^
**Fert x myc**	**4**	*p* = 0.1837	*p* = 0.0213	*p* = 0.5222	*p* = 0.0033	*p* = 3.42e^–12^

### Plant Biomass Production

**FIGURE 2:**
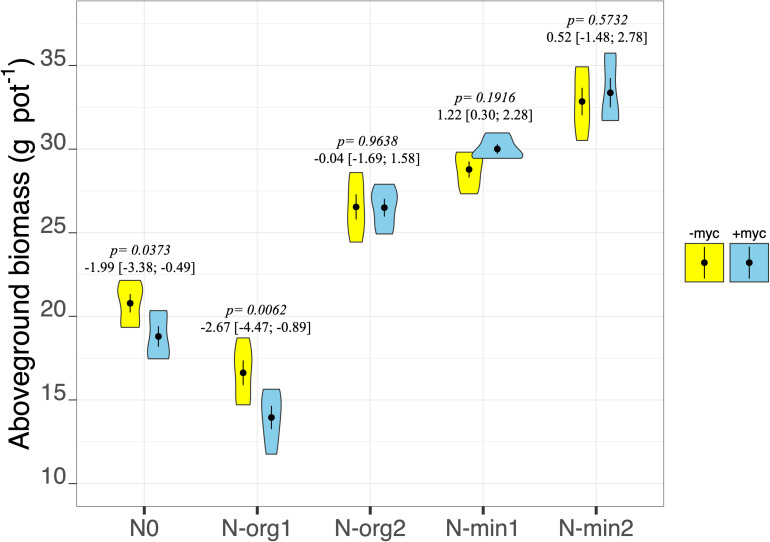
Aboveground biomass dry weight of durum wheat in the different fertilization treatments in the absence (–myc) or presence (+myc) of arbuscular mycorrhizal (AM) fungal inoculum. Circles inside plots represent means, with whiskers representing ± SE (*n* = 5). The width of the plot shows the density distribution of values. *p*-values for pairwise comparisons, Δ means, and estimated 95% confidence intervals (CIs) in brackets between +myc and –myc within the same fertilization treatment are reported above the plots.

**FIGURE 3:**
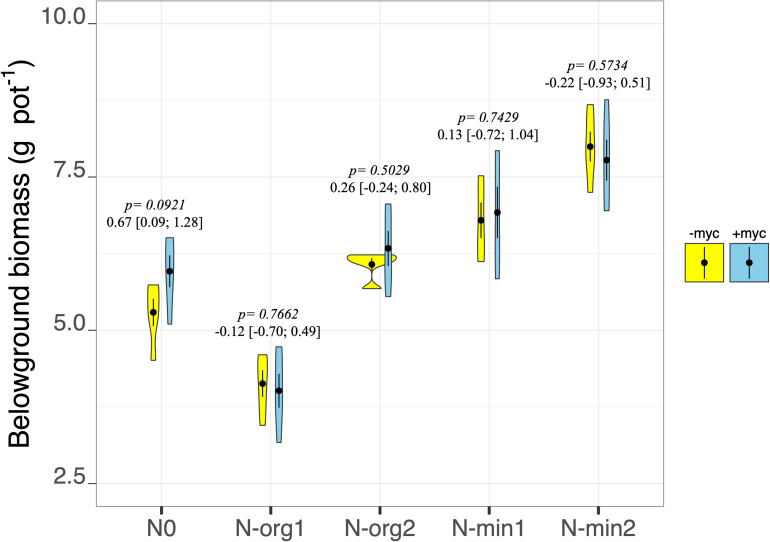
Belowground biomass dry weight of durum wheat in the different fertilization treatments in the absence (–myc) or presence (+ myc) of arbuscular mycorrhizal (AM) fungal inoculum. Circles inside plots represent means, with whiskers representing ± *SE* (*n* = 5). The width of the plot shows the density distribution of values. *p*-values for pairwise comparisons, Δ means, and estimated 95% confidence intervals (CIs) in brackets between +myc and –myc within the same fertilization treatment are reported above the plots.

Fertilization had pronounced effects on plant biomass production in both the above- and belowground fractions (fertilization treatment *p* < 2e^–16^ and *p* = 9.85e^–15^, respectively, for above- and belowground biomass; Table 2 and Figures 2, 3). Mineral N fertilization always increased biomass production compared to the non-fertilized treatment (N0). In particular, increases of 48 and 67% (overall means of +myc and −myc) were observed in aboveground biomass production in N-min1 and N-min2, respectively, compared to N0 (Figure 2). The same trend was observed for belowground biomass production: increases of 21 and 40% in N-min1 and N-min2, respectively, compared to N0 (Figure 3). In contrast, different responses were observed for the addition of the two organic sources (N-org1 and N-org2). Increases of 34 and 10% in above- and belowground biomass, respectively, were observed in N-org2 compared to N0, whereas decreases of 23 and 28%, respectively, were observed in N-org1 compared to N0 (Figures 2, 3).

The interaction of AM fungal inoculation and fertilization treatment had strong effects on aboveground biomass (fertilization × AM fungal inoculation interaction *p* = 0.0213; Table 2 and Figure 2). Strong effects of the presence of AM fungi on aboveground biomass production were observed for N0 and N-org1 (pairwise comparisons of +myc and –myc for N0 and N-org1, respectively, *p* = 0.0373 and *p* = 0.0062; Figure 2), although no noticeable difference ascribable to AM fungal inoculation was observed in N-min1, N-min2, or N-org2 compared to the respective non-inoculated treatments (all *p* values > 0.19; Figure 2). Specifically, the presence of AM fungi led to decreases of 9.5 and 16% in aboveground biomass production for N0 and N-org1, respectively, compared to the same non-inoculated fertilization treatments. AM fungal inoculation did not affect belowground biomass (AM fungal inoculation treatment *p* = 0.4090; interaction between the two main factors *p* = 0.5222; Table 2 and Figure 3). However, the presence of the AM fungal inoculum increased the total root length and soil root density (AM fungal inoculation treatment *p* = 0.0119 and *p* = 0.0118 for total root length and soil root density, respectively; Table 3). Also, the root-to-shoot ratio was affected by AM fungal inoculation, however, its effects on this parameter were dependent on the fertilization treatment (fertilization × AM fungal inoculation interaction *p* = 0.0414; Table 3). In particular, the values increased from 0.26 ± 0.013 to 0.25 ± 0.007 in the absence of the inoculum to 0.32 ± 0.015 and 0.29 ± 0.025 in the presence of AM fungi for N0 and N-org1, respectively (*p* = 0.0018 and *p* = 0.0423 for N0 and N-org1, respectively; Table 3). Again, no detectable difference ascribable to AM fungal inoculation was observed in N-min1, N-min2, and N-org2 compared to the respective non-inoculated treatments (all *p* values > 0.60; Table 3).

**TABLE 3:** Total root length, soil root density (SRD), specific root length (SRL), root-to-shoot ratio (R:S), aboveground biomass N concentration, and total mineral N residual in soil in the different fertilization treatments in the absence (–myc) or presence (+ myc) of arbuscular mycorrhizal (AM) fungal inoculum.

		**Root length**	**SRD**	**SRL**		**Nitrogen**	**Soil mineral N**
		**(m)**	**(cm g^–1^)**	**(m g^–1^)**	**R:S**	**concentration (%)**	**(mg kg^–1^)**
N0	**-myc**	730 (±23)*^*p = 0.0203*^*	9.74 (±0.31)*^*p = 0.0204*^*	138.3 (±2.95)*^*p = 0.5978*^*	0.26 (±0.014)*^*p = 0.0018*^*	0.63 (±0.01)*^*p = 0.0861*^*	2.61 (±0.10)*^*p*^*^<^*^0.0001^*
	**+myc**	844 (±23)	11.26 (±0.30)	142.5 (±5.91)	0.32 (±0.016)	0.55 (±0.01)	2.02 (±0.08)
*Δ means and estimate 95% CIs*		114.0 (68.6; 184.0)	1.52 (0.91; 2.45)	4.16 (−6.67; 15.90)	0.06 (0.03; 0.10)	−0.08 (−0.10; −0.06)	−0.59 (−0.81; −0.35)
N-org1	**-myc**	570 (±33)*^*p = 0.9974*^*	7.61 (±0.43)*^*p = 0.9966*^*	138.2 (±4.05)*^*p = 0.5504*^*	0.25 (±0.007)*^*p = 0.0423*^*	0.68 (±0.01)*^*p = 0.0321*^*	2.01 (±0.03)*^*p = 0.8846*^*
	**+myc**	568 (±26)	7.58 (±0.34)	143.0 (±6.03)	0.29 (±0.025)	0.79 (±0.01)	2.02 (±0.03)
*Δ means and estimate 95% CIs*		−1.6 (−72.9; 72.7)	−0.02 (−0.97; 0.97)	4.85 (−10.20; 15.60)	0.04 (0.00; 0.10)	0.11 (0.08; 0.14)	0.01 (−0.08; 0.08)
N-org2	**-myc**	939 (±5)*^*p = 0.3815*^*	12.52 (±0.70)*^*p = 0.3825*^*	154.7 (±2.79)*^*p = 0.7086*^*	0.23 (±0.008)*^*p = 0.6019*^*	0.74 (±0.02)*^*p = 0.0066*^*	2.73 (±0.04)*^*p = 0.0002*^*
	**+myc**	992 (± 29)	13.22 (±0.38)	157.3 (±5.71)	0.24 (±0.007)	0.89 (±0.04)	2.27 (±0.07)
*Δ means and estimate 95% CIs*		53.1 (−14.9; 93.6)	0.71 (−0.20; 1.25)	2.57 (−6.23; 16.90)	0.01 (−0.01; 0.03)	0.15 (0.08; 0.25)	−0.46 (−0.57; −0.28)
N-min1	**-myc**	957 (±58)*^*p = 0.1561*^*	12.76 (±0.78)*^*p = 0.1552*^*	142.1 (±10.64)*^*p = 0.3164*^*	0.24 (±0.012)*^*p = 0.7540*^*	0.76 (±0.05)*^*p = 0.0009*^*	2.15 (±0.09)*^*p = 0.0984*^*
	**+myc**	1,042 (± 54)	13.90 (±0.72)	151.4 (±6.48)	0.23 (±0.013)	0.95 (±0.04)	1.99 (±0.05)
*Δ means and estimate 95% CIs*		84.9 (−53.8; 219.0)	1.13 (−0.71; 2.92)	9.26 (−8.70; 35.30)	−0.01 (−0.04; 0.02)	0.19 (0.07; 0.30)	−0.16 (−0.35; 0.01)
N-min2	**-myc**	1,157 (±32)*^*p = 0.2511*^*	15.42 (±0.42)*^*p = 0.2519*^*	144.9 (±4.21)*^*p = 0.0576*^*	0.24 (±0.010)*^*p = 0.6019*^*	1.13 (±0.08)*^*p = 0.0497*^*	2.41 (±0.08)*^*p = 0.3922*^*
	**+myc**	1,243 (±53)	16.58 (±0.70)	160.2 (±5.11)	0.23 (±0.012)	1.26 (±0.06)	2.32 (±0.09)
*Δ means and estimate 95% CIs*		86.5 (−6.8; 209.0)	1.15 (−0.09; 2.79)	15.3 (4.53; 28.10)	−0.01 (−0.03; 0.01)	0.13 (−0.05; 0.30)	−0.09 (−0.29; 0.11)

Source	**df**						

Fert	**4**	*p* < 2e^–16^	*p* < 2e^–16^	*p* = 0.0309	*p* = 0.0004	*p* < 2e^–16^	*p* = 1.03e^−07^
Myc	**1**	*p* = 0.0119	*p* = 0.0118	*p* = 0.0522	*p* = 0.0269	*p* = 0.0001	*p* = 3.32e^−06^
Fert × myc	**4**	*p* = 0.5457	*p* = 0.5493	*p* = 0.8006	*p* = 0.0414	*p* = 0.0055	*p* = 0.0009

*Data are means (*n* = 5), with 1 *SE* in parentheses. *p*-values for pairwise comparisons within the same fertilization treatment are reported as superscripts. Δ means and estimated 95% confidence intervals (CIs) in brackets between +myc and −myc are reported below each fertilization treatment. ANOVA results and *p*-values for the main factors and their interactions are reported below each response variable.*

### Plant Biomass Nitrogen Concentration, Uptake and Recovery, and Nitrogen in Soil

**FIGURE 4:**
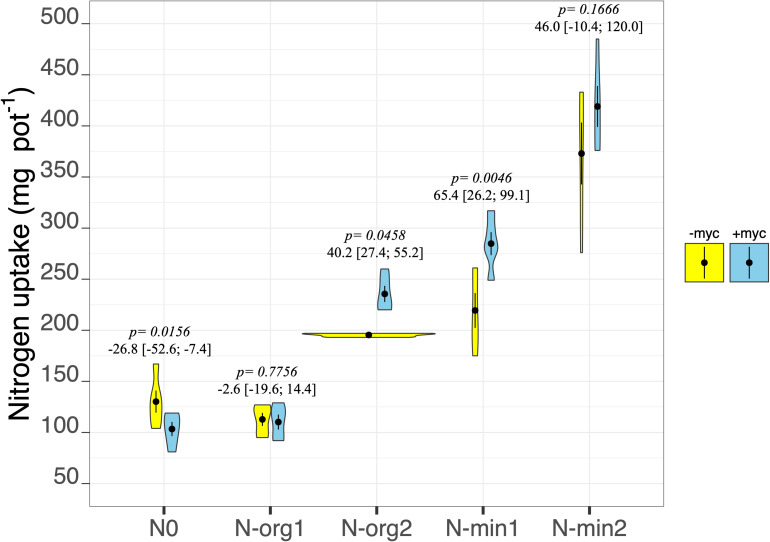
N uptake of durum wheat in the different fertilization treatments in the absence (–myc) or presence (+myc) of arbuscular mycorrhizal (AM) fungal inoculum. Circles inside plots represent means, with whiskers representing ± *SE* (*n* = 5). The width of the plot shows the density distribution of values. *p*-values for pairwise comparisons, Δ means, and estimated 95% confidence intervals (CIs) in brackets between +myc and –myc within the same fertilization treatment are reported above the plots.

Fertilization strongly affected the N concentration in aboveground biomass (fertilization treatment *p* < 2e^–16^; Table 3), showing the highest values in N-min2 (overall mean of +myc and −myc = 1.19% ± 0.051%) and the lowest in N0 (overall mean of +myc and −myc = 0.59% ± 0.013%). We detected an interaction between AM inoculation and fertilization treatment on N concentration (fertilization × AM fungal inoculation interaction *p* = 0.0055; Table 3). AM fungal inoculation decreased the N concentration in N0, although increases were observed in all other fertilization treatments (Table 3). The same trend was observed for N uptake, except in N-org1, where no difference due to inoculation was observed. Increases in N uptake due to the presence of AM fungi were observed in N-org2, N-min1, and N-min2; a decrease of 16.5% (*p* = 0.0156) was observed in N0, and no difference was observed in N-org1 (Figure 4). The values observed in N-org1 in either the presence or absence of AM fungi did not differ from those observed in N0 in the presence of AM fungi (112.8 ± 6.42, 110.2 ± 7.31, and 103.4 ± 6.93 mg of N acquired per pot in N-org1 –myc, N-org1 +myc, and N0 –myc, respectively). The same trend was observed for mineral N residual in soil (N-NH_4_^+^, N-NO_3_^–^, and N-NO_2_^–^) assayed at the end of the experiment (Table 3). In detail, the total mineral N in soil was 2.01 ± 0.03 and 2.02 ± 0.03 mg per kg of soil in N-org1 –myc and + myc, respectively, and 2.02 ± 0.08 in N0 in the presence of AM fungi. It is interesting that, for this parameter, N0 had a lower mineral N in soil in the presence of AM fungi, although plant N uptake was higher in the absence of the inoculum. A marked reduction in mineral N residual in soil ascribable to AM fungal inoculation was also observed in N-org2; no notable differences were found in N-min1 and N-min2, although lower average values were observed in both of these treatments (Table 3).

**FIGURE 5:**
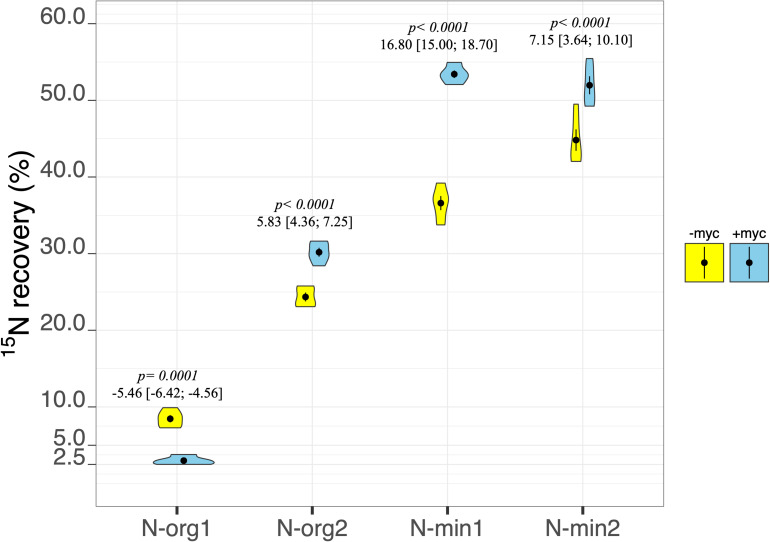
Percentage of N recovery of durum wheat in the different fertilization treatments in the absence (–myc) or presence (+myc) of arbuscular mycorrhizal (AM) fungal inoculum. Circles inside plots represent means, with whiskers representing ± *SE* (*n* = 5). The width of the plot shows the density distribution of values. *p*-values for pairwise comparisons, Δ means, and estimated 95% confidence intervals (CIs) in brackets between +myc and –myc within the same fertilization treatment are reported above the plots.

The percentage of plant biomass N derived from the fertilizer (%N_*rec*_) was strongly affected by the interaction between fertilization treatment and AM fungal inoculation (*p* ≤ 0.001; Table 2 and Figure 5). In particular, a marked increase in N recovery ascribable to the presence of AM fungi was observed in N-org2, N-min1, and N-min2 (Figure 5). By contrast, in N-org1, AM fungal inoculation decreased the percentage of N derived from the organic source from an average of 8.45% to an average of 2.99% (Figure 5).

## Discussion

In the present experiment, the degree of mycorrhization varied with respect to the availability of N in the substrate, having the highest observed values in N0 and the lowest ones in N-min2. This is in agreement with the findings of Ercoli et al. (2017) for durum wheat under field conditions and the results of Gamper et al. (2004) and Staddon et al. (2004), who found a reduction in mycorrhizal root colonization in plants grown in nutrient-rich soils. In nutrient-rich conditions, the plant is better able to satisfy its nutritional needs without having to transfer photosynthates to the mycorrhizae, whereas in N-deficient conditions, the plant is stimulated to develop a symbiotic relationship to increase its chances of intercepting more nutrients. However, in this experiment, despite the fact that in the N-org1 treatment, N availability was extremely low for plants, we found that the percentage of mycorrhization in N-org1 was lower compared to the treatment N0. This could be connected to the poor development of the root system in N-org1 compared to the non-fertilized treatment or to the different timing in soil N availability between the two treatments (as discussed later). Further research should be conducted to elaborate on this aspect.

In the present research, the increase in the percentage of mycorrhization was not associated with a corresponding benefit in terms of plant growth and N uptake. In fact, in the absence of fertilization (N0), mycorrhization resulted in reduced plant growth, N concentration, and uptake, which shows how AM fungi strongly compete for the little available N, whereas small benefits were sometimes observed when N fertilizer was applied. In fact, when N was not a limiting factor, mycorrhizal colonization resulted in an increase in N concentration in plant tissue and overall N uptake. Indeed, contrasting results for the relationship between AM fungal root colonization and its effects on plant performance have been reported (e.g., van der Heijden et al., 2006; Büscher et al., 2012; Corrêa et al., 2014; Fellbaum et al., 2014). However, in our experiment, mycorrhization led to a more efficient acquisition of soil N regardless of plant growth, leaving less residual N in the soil. In particular, it is interesting that N0 had a lower mineral residual N in the soil in the presence of AM fungi, although plant N uptake was higher in the absence of the inoculum. This suggests that AM fungi have efficiently used the limited amount of soil N for their own growth. This behavior has been previously reported, as AM fungi growing in N-limiting conditions regulate the expression of ammonium transporters, which acquire N from the soil retaining the N for their own metabolism (Guether et al., 2009b; Pérez-Tienda et al., 2011).

The use of organic matter with a high C:N ratio (N-org1) had clear detrimental effects on biomass production; in this treatment, mycorrhization amplified these effects and reduced N recovery from organic matter, however, mycorrhization increased the N concentration so that it did not influence overall N uptake. These results partially contrast with the findings of Saia et al. (2014) who observed that the presence of AM fungi in organic matter amended soil increased plant growth and N uptake, while markedly reducing N recovery from organic matter compared to an uninoculated control. In their experiment, however, the availability of mineral N in the substrate and the amount of organic matter added were clearly higher than in our experiment. To understand our results, one should remember that the decay of organic matter with a high C:N ratio is characterized by two phases: N net immobilization followed by N net mineralization (Tian et al., 1992; Quemada and Cabrera, 1995). Initial conditions of N deficiency, realized immediately after the application of organic matter, could have increased the competition between the plant and AM fungi, strongly inhibiting the plant’s early growth. In fact, AM fungi have a high N demand for their metabolism (Hodge and Fitter, 2010) and when soil N is deficient can compete with the host plant for the available N (Püschel et al., 2016; Treseder and Allen, 2002), which affects biomass production. The increased N was subsequently used by the plant (also transferred by AM fungi to the plant) to increase the concentration of the element in tissue without increasing plant growth. However, at the same time, our results support the notion that, in N-org1, the two symbionts could have taken up N from different soil N pools. In fact, AM fungi can influence the mineralization process and use the nutrients derived from it through their direct interaction with soil microorganisms involved in the soil N cycle (Veresoglou et al., 2012; Hodge and Storer, 2015; Bukovská et al., 2018). Although AM fungi are unable to mobilize organically bound nutrients (Bunn et al., 2019), they can stimulate microbial decomposition through the release of labile C compounds in their hyphosphere, increasing the decomposition of organic residues (Talbot et al., 2008; Paterson et al., 2016). AM fungi could have more efficiently used the N from the organic matter as an N source, leaving the original substrate N supply for the plant. Indeed, mycorrhization did not affect the overall plant N uptake in N-org1, although it strongly decreased N recovery from the organic matter.

In N-org2, AM fungi increased all plant N-related parameters (N concentration, N uptake, and N recovery) without affecting plant biomass production. The difference observed between the two organic treatments could be ascribed to the lower C:N ratio of the organic patch in N-org2 compared to N-org1 (16.2 vs. 49.7 C:N ratio in N-org2 and N-org1, respectively). In fact, contrary to what was described in N-org1, the mineralization of an organic patch with a relatively low C:N ratio (as in N-org2) can reduce the competition between the plant and soil microorganisms, releasing a substantial amount of N to sustain the growth of both plant and fungi (Hodge et al., 2000). However, in N-org2, mycorrhization increased N recovery, consistent with other pot experiments in which AM fungi transferred a substantial amount of N derived from an organic patch to the host plant without affecting plant biomass (Hodge et al., 2001; Herman et al., 2012). However, here it is important to highlight the fact that the absolute amount of N supplied was higher in N-org2 than in N-org1 (118.3 vs. 362.7 mg N per pot in N-org1 and N-org2, respectively). This could also have affected the total amount of N available in the soil and therefore the competition between the plant and soil organisms, including AM fungi.

In the N mineral fertilization treatment, AM inoculation resulted in no increase in biomass (either above- or belowground) despite an increase in N-related parameters in plant tissue. Reynolds et al. (2005) hypothesized that the AM fungal C drain imposed on the plant prevents the increased plant growth that is usually observed when more N is available. As observed by Bago et al. (2000), the amount of carbohydrates transferred to the symbiont fungi can be up to 20% of total plant C assimilation. However, the higher N concentration in the biomass, although it does not influence biomass production, could influence the yield quality of the grain at maturity, which highlights the potential impact of soil microbiota on food quality (Rillig et al., 2018). In fact, in wheat plants, the amount of N in the grain is directly related to the amount of N accumulated in the biomass at anthesis and remobilized (Hirel et al., 2007). Moreover, the increment in aboveground biomass N concentration could affect the plant’s photosynthetic capacity since RuBisCO (ribulose-1,5-bisphosphate carboxylase/oxygenase), the primary CO_2_-fixing enzyme in C3 plants, accounts for as much as 75% of leaf N (Chapin et al., 1987) with positive consequences on plant performance and climate change mitigation.

Arbuscular mycorrhizal fungal inoculation increased the root-to-shoot ratio in N0 and N-org1 only. This increase was due to the detrimental effects observed in aboveground biomass rather than the positive effects on growth in belowground biomass. It is possible that AM fungi in N0 and N-org1 altered the C allocation within the plant tissue, increasing the amount of C transferred to the root system and potentially to themselves. Indeed, results from an experiment using ^14^C showed that AM fungi can have strong C-sink effects modulating C allocation among plant tissue in a symbiosis involving barley and *Glomus mosseae* (now *Funneliformis mosseae;* Lerat et al., 2003). In the other treatments (N-min1, N-min2, and Norg-2), the higher aboveground biomass compared to N0 and N-org1 presumably allowed for sufficient photosynthate to satisfy the fungal requirements and those for plant growth, thereby reducing the C partitioning effect.

Our results suggest that mycorrhization can (indirectly) influence the mineralization of organic matter and that the magnitude of this effect varies by type of organic matter. In fact, when the organic matter had a low C:N ratio, the presence of AM fungi favored mineralization processes and, consequently, plant N uptake; in contrast, when the organic matter had a high C:N ratio, we observed a clear reduction in N recovery from organic matter, which suggests that, under N-limiting conditions, the presence of AM fungi can have pronounced effects on competition for different N sources among plants, microorganisms, and AM fungi themselves. The increase in N recovery from the substrate ascribed to the presence of AM fungi (except in N-org1) would certainly have positive agro-environmental implications, as it would reduce the risk for N leaching. The opposite effect observed in N-org1 is of little concern, as here, N is immobilized in organic matter with a high C:N ratio and/or in the AM fungal biomass, and hence there is comparatively little risk of it being released into the soil.

In conclusion, our results reveal that the effects of AM fungi on plant performance are driven by the nature and availability of N in soil and that, even when mycorrhization does not affect plant biomass production, AM fungi can influence the quality of agricultural products by increasing the uptake of N. At the same time, our research shows an active role of mycorrhizae in favoring N recovery from the substrate, which will surely increase the sustainability of the agroecosystem by reducing the risk for N loss. The present study contributes to knowledge on the effects of AM fungi in N uptake and recovery, which is required to make the best use of mycorrhizal technology to help achieve sustainable intensification (Rillig et al., 2016). Finally, considering that different AM fungal taxa have different functional responses to soil N availability (Treseder et al., 2018) and that, in the present experiment, we used a mix of two AM fungi species both belonging to the Glomeraceae family, further research is needed to increase knowledge about the functionality of the complex mycorrhizal community living in agroecosystems.

## Data Availability Statement

The datasets generated for this study are available on request to the corresponding author.

## Author Contributions

RI, DG, GA, and AF conceptualized and elaborated the data. DG, GA, and AF acquired the funds to conduct the experiment. RI carried out the formal analysis and wrote the first draft of the manuscript. DG, GA, AF, MS-H, and MR collaborated on the ideas and contributed critically to the drafts. All authors gave the final approval for publication.

## Conflict of Interest

The authors declare that the research was conducted in the absence of any commercial or financial relationships that could be construed as a potential conflict of interest.
