# Supplementary material for: Nitrogen Type and Availability Drive Mycorrhizal Effects on Wheat Performance, Nitrogen Uptake and Recovery, and Production Sustainability
Source: Front Plant Sci. 2020 Jun 19;11:760. doi: 10.3389/fpls.2020.00760 (PMC7318877; doi:10.3389/fpls.2020.00760)
Supplement: Supplementary file 1 [file Presentation_1.pdf]

# Supplementary Material

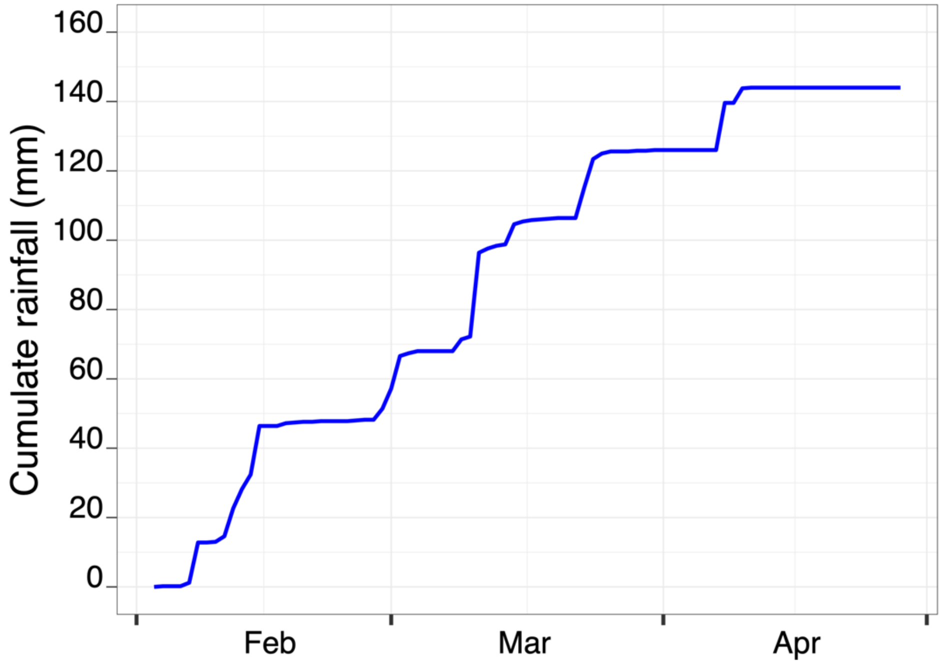


*Fig. S1*. Accumulated rainfall at the experimental site during the growing seasons

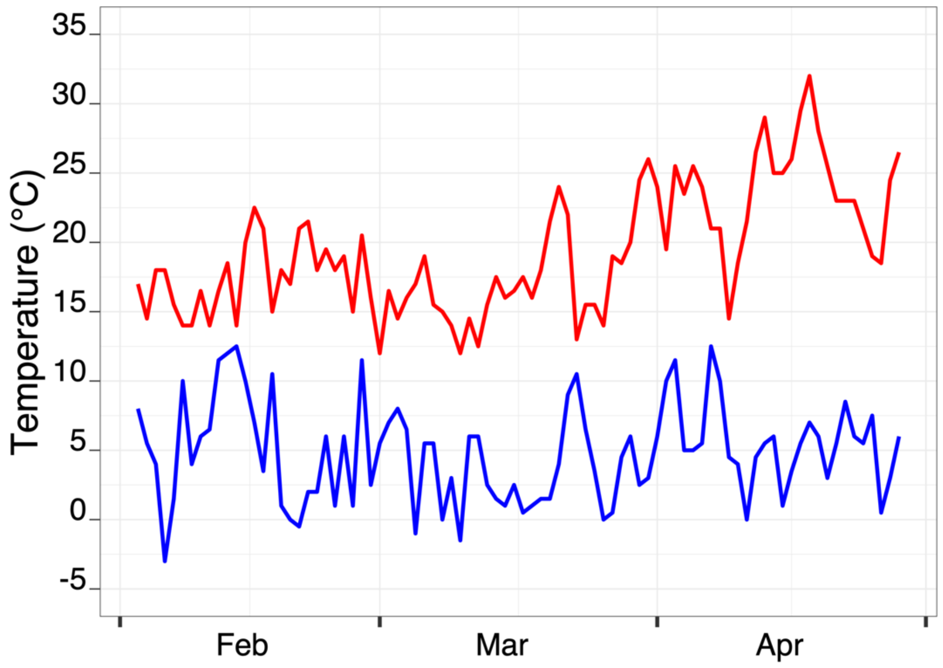


*Fig. S2*. Maximum (in red) and minimum (in blue) temperature at the experimental site during the growing seasons
